# Why do we keep reporting high-grade prostatic intraepithelial neoplasia (HGPIN)?

**DOI:** 10.1590/S1677-5538.IBJU.2016.02.02

**Published:** 2016

**Affiliations:** Conselheiro Internacional para América do Sul da Sociedade Internacional de Patologia Urológica (ISUP), Professor da Universidade de São Paulo (FMUSP), Director do Laboratório de Investigação Médica da FMUSP - LIM 55, Av. Dr. Arnaldo 455, Sao Paulo, Brazil, E-mail: katiaramos@uol.com.br; Universidade de São Paulo, Universidade de São Paulo (FMUSP), Sao Paulo, Brazil; Laboratório de Investigação Médica da FMUSP - LIM, Sao Paulo, Brazil

Over the years, concepts regarding prostate cancer (PC) have been changing tremendously.

Invasive prostate adenocarcinoma has a precursor, in situ lesion, as all other epithelial neoplasia, that McNeal and Bostwick described as “intraductal dysplasia” in 1984 (1) what we now call prostatic intraepithelial neoplasia (PIN).

At first, PIN was classified as I, II or III (2), being later simplified as low or high grade PIN (HGPIN). The latter was the only lesion that should be mentioned in the pathological report, since there was a close relation between HGPIN and invasive cancer (3).

At that point, prostate biopsy was routinely made in sextant with only six fragments taken randomly from the base, medium portion and apex of both sides of the prostate gland as suggested by Hogde in substitution to biopsy directed to nodules identified by rectal examination (4). Following HGPIN the chance of finding cancer was up to 35%, and a new biopsy was recommended after this diagnosis (5).

Different studies suggested that sextant biopsies were not adequate to search for prostate cancer and 12, 14, 16, 18 fragments and even saturation biopsies with over 30 cores taken in one biopsy section were proposed to correctly diagnose the disease. With the increased number of fragments, the diagnosis of HGPIN lost its power to predict PC in subsequent biopsies, with results similar to those found after a benign diagnosis (6). In addition, in the occasion of prostate cancer detection after a HGPIN diagnosis, radical prostatectomy shows favorable characteristics of the tumor (7).

An important change regarding PC was the review of the histological classification proposed by Gleason resulting in the termination of patterns 1 and 2 in biopsies in 2005 (8). Subsequently, the lowest histological grade in routine practice has been Gleason 6(3+3) that is now referred to as grade group 1 or ISUP grade 1 by a new classification proposed by a consensus meeting of the International Society of Urological Pathology (ISUP) on November 1st 2014 (9).

The Hopkins group published earlier a provocative paper proposing a discussion about the probable indolence of a prostate cancer Gleason 6(3+3). They suggested that PC Gleason 6(3+3) after the 2005 ISUP consensus, should not be called cancer considering that it is not related to unfavorable prognostic factors, does not metastasize to lymph nodes nor to distant organs after radical prostatectomy (10).

All the new data supported the management of PC that now prioritizes surveillance over treatment for well differentiated tumors. There are numbers of trials of active surveillance, now with a follow-up of 15 years, that show cancer specific survival of 100%, strengthening even more the Hopkins concept (11). Although there was a significant change in the comprehension and management of PC considering the well differentiated tumors we remain reporting HGPIN. For patients, the term “high grade neoplasia” is very alarming and a motive of great apprehension. Not infrequently, patients reach out to their doctors anxiously when facing this diagnosis in a pathology report, causing stress also to urologists and clinicians.

In addition, some of the lesions that in the past we used to call florid HGPIN are now being called intraductal cancer (IDC), a diagnosis frequently associated with high grade invasive cancer. It is now recommended that a diagnosis of IDC be associated with a note in the pathology report recommending a better attention to this particular patient (12).

Since HGPIN now only correlates with a marginal increase in the incidence of prostate cancer, I would like to propose that we pathologists begin to omit this diagnosis in our reports as we did with low grade PIN in the past. This will certainly reduce stress in patients and doctors.
